# Complete Genome Sequence of African Swine Fever Virus Isolated from a Domestic Pig in Timor-Leste, 2019

**DOI:** 10.1128/MRA.00263-21

**Published:** 2021-07-01

**Authors:** Patrick Mileto, Felisiano da Conceição, Vittoria Stevens, David Cummins, Andrea Certoma, Matthew J. Neave, Joanita Bendita da Costa Jong, David T. Williams

**Affiliations:** a CSIRO, Australian Centre for Disease Preparedness, Geelong, Victoria, Australia; b National Directorate of Veterinary Services of the Ministry of Agriculture and Fisheries, Government of Timor-Leste, Comoro, Dili, Timor-Leste; Queens College CUNY

## Abstract

Here, we report the complete genome sequence of the African swine fever virus (ASFV) isolate ASFV/Timor-Leste/2019/1, isolated from a domestic pig during the first outbreak of ASF in Timor-Leste in 2019. Using target enrichment short-read Illumina data combined with long-read Oxford Nanopore data, we assembled a full-length genome sequence of 192,237 bp.

## ANNOUNCEMENT

African swine fever virus (ASFV) is a double-stranded DNA virus belonging to the genus *Asfivirus*, family *Asfarviridae*. It has a genome sequence approximately 170 to 194 kb long, encoding between 150 and 167 open reading frames. ASFV causes a devastating hemorrhagic disease in domestic and wild suids (1). Following its introduction into China in 2018, ASF spread rapidly through Southeast Asia (2) and emerged in Timor-Leste in September 2019 (3, 4).

Samples collected from sick and dying pigs in the Dili municipality were sent to the Australian Centre for Disease Preparedness for diagnostic testing and molecular characterization. An ASFV isolate was obtained from a nasal-rectal swab following inoculation onto primary porcine bone marrow cells and confirmed by ASFV PCR and specific immunofluorescence assay (5, 6). ASFV DNA was extracted from infectious tissue culture supernatant using the Applied Biosystems MagMAX-96 viral RNA kit (Thermo Fisher Scientific). A Nextera XT library (Illumina) was prepared using 2 ng of DNA extract, followed by ASFV-specific genome enrichment using the myBaits target capture kit (Arbor Biosciences), following the manufacturer’s instructions. The prepared library was then sequenced on an Illumina MiSeq instrument using the MiSeq reagent kit v.2 (150-bp paired-end [PE] reads).

To resolve the terminal repeats, the 3′ terminal repeat of the virus genome was amplified and the product sequenced using MinION long-read sequencing (Oxford Nanopore technology). The forward primer was designed to bind upstream of the 3′ terminal repeat (ASF_F_right: 5′-GTATTTACGGTAGGGTTTATTACCG), and the reverse primer bound slightly upstream of the terminal 3′ hairpin sequence (ASF_HP: 5′-GCAGGTACAATTTTATTATATAGTGCAG). The region was amplified using the HotStarTaq master mix kit (Qiagen) with the following thermocycler conditions: 95°C for 15 min; 5 cycles of 95°C for 45 s, 40°C for 45 s, and 72°C for 2 min; 30 cycles of 95°C for 45 s, 55°C for 45 s, and 72°C for 2 min; and then a final extension of 72°C for 7 min. Oxford Nanopore libraries were prepared using the PCR barcoding kit (SQK-PBK004) and sequenced on an R9.4.1 flow cell (FLO-MIN106) for 48 h.

Default parameters were used for all software cited unless otherwise specified. For the genome assembly, the Illumina reads were mapped to the ASFV China/2018/AnhuiXCGQ genome (GenBank accession number MK128995.1) using the Geneious v.11.1.4 mapping algorithm and a draft consensus was generated. To complete the terminal repeats, the adapters were trimmed from the Oxford Nanopore reads using Porechop v.0.2.1 (7), and reads shorter than 1,360 bp and larger than 1,375 bp were removed using NanoFilt v.2.3.0 (8). The filtered reads were aligned using MAFFT v.7.407 (9) and imported into Geneious v.11.1.4 to generate a consensus of the repeat region. The consensus sequence was then amended to the previously generated Illumina genome sequence and refined by mapping the Illumina data and removing any errors in Geneious v.11.1.4. The final assembly was 192,237 bp long with 76× Illumina coverage and 1,367× Oxford Nanopore coverage of the terminal repeat, and it had a GC content of 38.3% (Table 1). The completed genome sequence was annotated by transferring the gene features from China/2018/AnhuiXCGQ in Geneious v.11.1.4. A total of 178 predicted proteins were annotated.

**TABLE 1:** Summary details of the ASFV/Timor-Leste/2019/1 genome sequence

Isolate name	Genome size (bp)	GC content (%)	No. of ORFs^*a*^	No. of Illumina reads	No. of mapped Illumina reads	No. of Oxford Nanopore reads	No. of mapped Oxford Nanopore reads	GenBank accession no.	BioProject accession no.
ASFV/Timor-Leste/2019/1	192,237	38.3	178	226,084	111,527	31,493	5,152	MW396979	PRJNA725035

a ORFs, open reading frames.

The ASFV/Timor-Leste/2019/1 genome contains 17 single nucleotide polymorphisms (SNPs) compared to China/2018/AnhuiXCGQ. Sequence analysis indicated that ASFV/Timor-Leste/2019/1 belongs to p72 genotype II, CD2v serogroup 8, IGR variant II, and CVR 1 subtype, characteristic of European, Chinese, and Southeast Asian viruses (10–13).

### Data availability.

This assembled genome sequence has been deposited in NCBI GenBank under the accession number MW396979. The raw sequence data have been deposited in the NCBI Sequence Read Archive (SRA) database under the BioProject accession number PRJNA725035.
